# Changes in pulse waveforms in response to intraocular pressure elevation determined by laser speckle flowgraphy in healthy subjects

**DOI:** 10.1186/s12886-021-02070-7

**Published:** 2021-08-21

**Authors:** Chie Iwase, Takeshi Iwase, Ryo Tomita, Tomohiko Akahori, Kentaro Yamamoto, Eimei Ra, Hiroko Terasaki

**Affiliations:** 1 Department of Ophthalmology, Nagoya University Graduate School of Medicine, Nagoya, Japan; 2 Department of Ophthalmology, Akita University Graduate School of Medicine, 1-1-1 Hondou, Akita-city, Akita, 010-8543 Japan

**Keywords:** Laser speckle flowgraphy, Intraocular pressure, Elevation, Optic nerve head

## Abstract

**Background:**

The influences of intraocular pressure (IOP) elevations on the pulse waveform in the optic nerve head (ONH) were evaluated using laser speckle flowgraphy (LSFG) in normal subjects.

**Methods:**

This prospective cross-sectional study was conducted at the Nagoya University Hospital. An ophthalmodynamometer was pressed on the sclera to increase the IOP by 20 mmHg or 30 mmHg for 1 min (experiment 1, 16 subjects) and by 30 mmHg for 10 min (experiment 2, 10 subjects). The mean blur rate (MBR) and the eight pulse waveform parameters determined using LSFG were measured before, immediately after and during an IOP elevation, and after the IOP returned to the baseline pressure.

**Results:**

A significant elevation in the IOP and a significant reduction in the ocular perfusion pressure (OPP) were found after applying the ophthalmodynamometer (both, *P* < 0.001). The blowout score (BOS) reduced significantly (*P* < 0.001), and the flow acceleration index (FAI; *P* < 0.01) and resistivity index (RI; *P* < 0.001) increased significantly immediately after increasing the IOP by 20 or 30 mmHg (experiment 1). The BOS reduced significantly (*P* < 0.001), and the FAI (*P* < 0.01) and RI (*P* < 0.001) increased significantly after the IOP elevation by 30 mmHg in both experiment 2 and 1. However, the BOS and RI recovered significantly at time 10 compared to that in time 0 (immediately after IOP elevation) during the 10-min IOP elevation (*P* < 0.001 and *P* = 0.008, respectively).

**Conclusions:**

In conclusion, the BOS, FAI, and RI of the pulse waveforms changed significantly with an acute elevation in the IOP. The change should be related to the larger difference between the maximum and minimum MBRs during the IOP elevation.

## Introduction

One method to study the blood flow regulation has been to change artificially the ocular perfusion pressure (OPP) [1–4]. The OPP is defined as the difference between the arterial and venous pressure in a vascular bed. This reduced OPP should then result in a reduction of the ocular blood flow. In the eye, the venous pressure can reach high levels when the intraocular pressure (IOP) is elevated, and it has to be slightly higher than the IOP to maintain sufficient outflow of the blood [5].

The effect of changes in the IOP on the ocular blood flow has been investigated by various methods mainly in laboratory experiments on different animal species [6–9], and the results have shown that the blood supply to the optic nerve head (ONH) was well autoregulated to maintain a constant blood flow despite changes in the IOP and OPP [10–15]. Recently, a study by Popa-Cherecheanu et al. has investigated the regulation of ocular blood flow under artificial continuous IOP elevation using a suction cup and laser Doppler flowmetry [16]. Earlier, a testing protocol was also established to examine the response of blood flow on the ONH induced by changes in the OPP induced by an artificial elevation of the IOP, and the elevated IOP was found to be stable and reproducible [1, 2].

Laser speckle flowgraphy (LSFG, Softcare, Fukutsu, Japan) can evaluate the ocular blood flow noninvasively and quickly [17–20]. An update of the software embedded in the most recent LSFG analyzer (LSFG Analyzer, v. 3.1.6;) has allowed us to record synchronized images from each cardiac cycle and determine various pulse waveform parameters which can be a new biomarker to detect and evaluate vascular diseases. The results of earlier studies have shown that the relationship between these waveform parameters and other variables e.g., age [21–24], mean intima–media thickness [25], and normal-tension glaucoma [21]. Accordingly, these pulse waveform parameters should be able to provide new information about the ONH autoregulation in response to OPP changes induced by artificial IOP elevation.

Thus, this study aimed to evaluate the influences of IOP elevations on the pulse waveform on the ONH using LSFG in normal subjects. To accomplish this, different parameters of the pulse waveform determined using LSFG were measured before, immediately after an elevation of the IOP, and after the IOP was returned to the baseline level.

## Methods

All the study participants were asked to abstain from alcoholic and caffeinated beverages on the morning of the examination. The pupil was dilated using 0.4% tropicamide/phenylephrine (Mydrin P; Santen Pharmaceutical Co., Ltd., Osaka, Japan) 30 min before the examinations, and the subjects rested in a quiet dark room for 10–15 min before the measurements to achieve stable hemodynamic conditions. All examinations were performed in the sitting position at approximately 12:00 h to avoid diurnal variations [26, 27]. The axial lengths were measured using partial optical coherence interferometry (IOLMaster; Carl Zeiss Meditec, La Jolla, CA), and the IOP was measured using a handheld tonometer (Icare; TiolatOy, Helsinki, Finland). The systolic blood pressure (SBP) and diastolic blood pressure (DBP) were measured using an automatic sphygmomanometer (CH-483C; Citizen, Tokyo, Japan). The mean arterial blood pressure (MAP) and OPP were calculated as follows:

MAP = DBP + 1/3(SBP – DBP);

OPP = 2/3MAP – IOP.

### Exclusion criteria

The exclusion criteria included the following: the best-corrected visual acuity of the eyes was less than 20/20, a history of ophthalmic or systemic disorders including glaucoma, diabetes, hypertension, and arrhythmia; a history of treatment, e.g., ocular laser or incisional surgery in both eyes; SBP of more than 150 mmHg; DBP of more than 90 mmHg; axial length of more than 27.0 mm; medical conditions that could influence the hemodynamics of the eye, e.g., vascular diseases, and a regular smoking habit.

### Experimental elevation of IOP

An ophthalmodynamometer (Inami, Tokyo, Japan) was used to apply pressure on the eye to increase the IOP after a topical anesthesia (0.4% Benoxil ophthalmic solution; Santen pharmaceutical co. ltd, Osaka, Japan). The device was pressed perpendicularly to the globe to make a fixed external pressure on the sclera [1]. Only the right eye was used for the experiment. The scale on the ophthalmodynamometer showed the force applied to the eye to increase the IOP. The IOP was measured during the application of the pressure using the Icare tonometer (Icare®; Tiolat Oy, Helsinki, Finland) before, during, and after the application of the pressure.

### Experiment 1

**Fig. 1:**
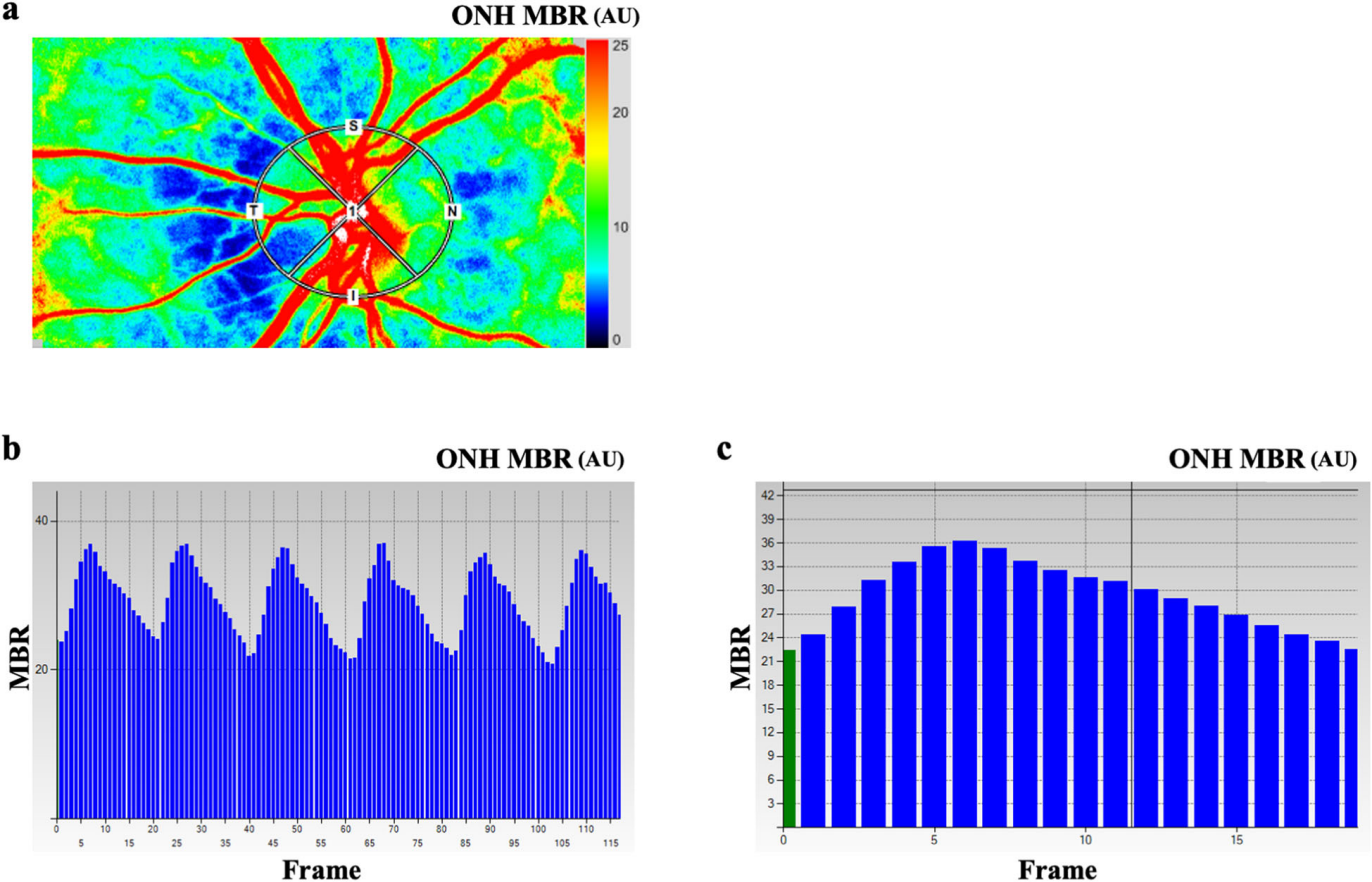
Analysis of the pulse waveform at the optic nerve head (ONH) using laser speckle flowgraphy (LSFG). Representative color-coded composite map (**a**). The ONH, the mean blur rate (MBR), and other waveform parameters can be measured within the marked circle. Pulse waves showing MBR changes due to the cardiac cycle for 4 s. The total number of frames is 118 in 1 scan (**b**). The change of the MBR in one heartbeat (**c**)

**Fig. 2:**
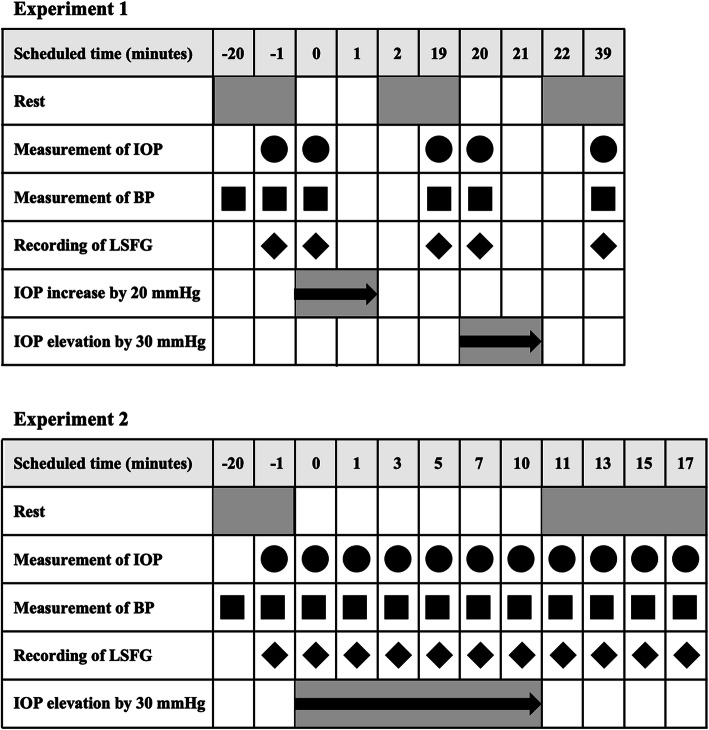
Illustration of the time course of the experiments

One experimenter placed the ophthalmodynamometer on the temporal sclera and another experimenter recorded the LSFG images (Fig. 1). First, the IOP was increased by 20 mmHg from the baseline for 1 min. After taking a rest for approximately 20 min, the IOP was increased by 30 mmHg from the baseline for 16 subjects. The LSFG images were recorded at 1 min before the IOP elevation (time − 1), 1 min after the elevation of the IOP by 20 mmHg (time 1), 18 min after the release of the pressure (time 19), 1 min after the elevation of the IOP by 30 mmHg (time 21), and 18 min after the release of the pressure (time 39) (Fig. 2).

### Experiment 2

The IOP was elevated by 30 mmHg from the baseline for 10 min using completely different subjects. The LSFG images were recorded before, immediately after the IOP elevation (time 0), and at 1 min (time 1), 3 min (time 3), 5 min (time 5), 7 min (time 7), and 10 min (time 10) while the IOP was elevated. Additionally, the LSFG images were recorded at 1 min (time 11), 3 min (time 13), and 5 min (time 15) after the release of the pressure on the eye (Fig. 2).

### Laser speckle flowgraphy (LSFG)

**Fig. 3:**
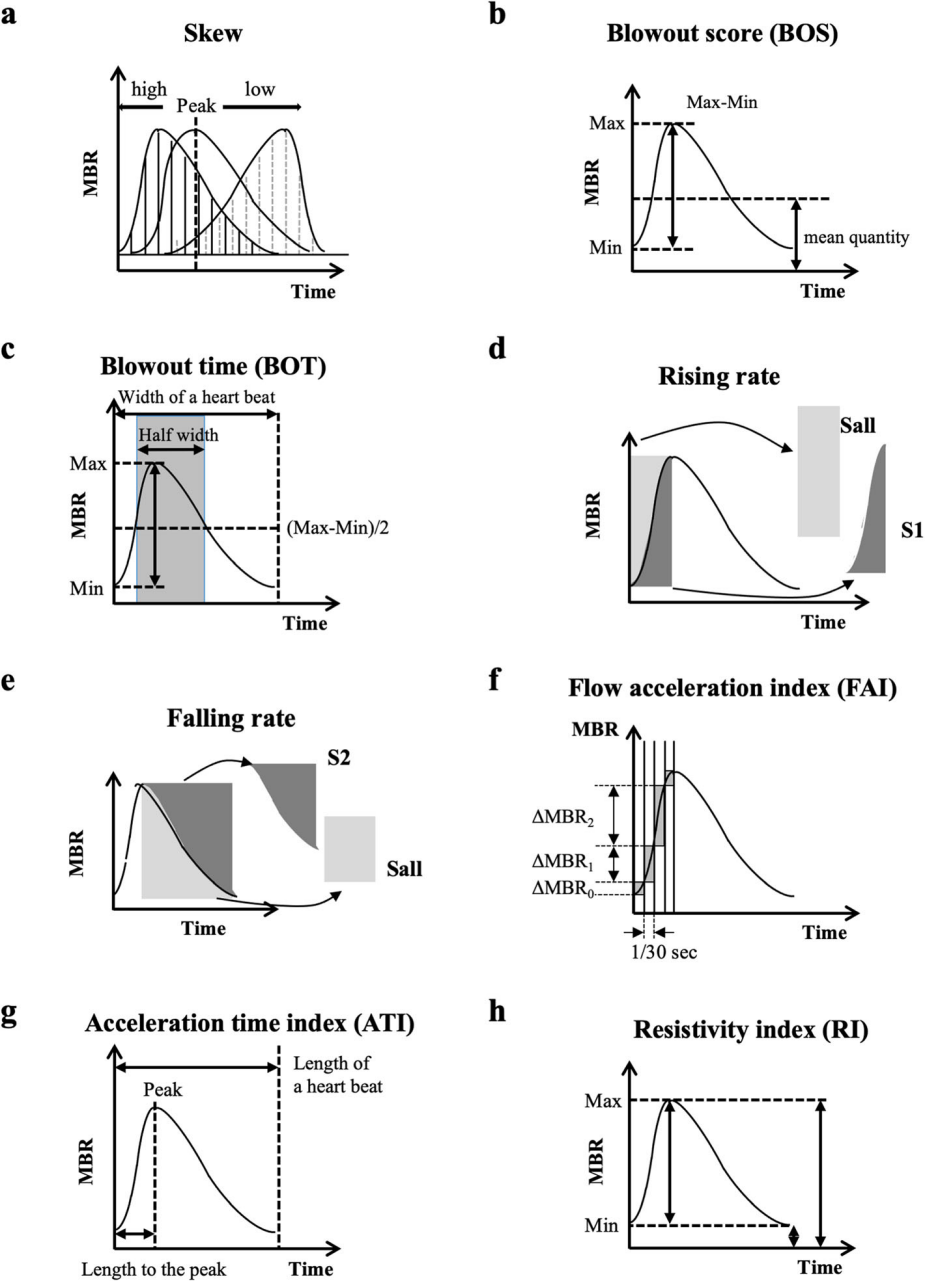
Pulse waveform analysis of the eight waveform parameters. The skewed shape shows the asymmetry of the waveform (**a**). If the shape of the waveform is symmetrical, the skew is 0 and if the peak comes earlier than that, the degree of skewness increases and the peak slower, and the degree of skewness decreases. The blowout score (BOS) indicates the amount of the blood flow volume in one heartbeat (width of a heartbeat) (**b**). The blowout time (BOT) represents the length of time that the wave maintained more than half of the mean of the maximum and minimum MBR in a heartbeat (**c**). The half-width is the duration of the time that the MBR is higher than (MBR max – MBR min)/2. The rising rate (RR) is the relationship of the area of the S1 to that of S all (**d**). S all is the square area before the peak and the S1 is the increasing area (d). The falling rate (FR) is the proportion of the area of the S2 to the S all (**e**). S all is the area of the square after the peak and the S2 is the decreasing area (**e**). The flow acceleration index (FAI) is the maximum change of the increasing MBR in 1/30 s (**f**). The acceleration time index (ATI) is the ratio of the duration of the time to reach peak (width to reach peak) in one heartbeat (width of a heartbeat) (**g**). The resistivity index (RI) is calculated by dividing the difference of MBR max and the MBR min by the MBR max (**h**)

The principles of LSFG have been described in detail [28–31]. The LSFG analyzer software separates the vascular and the tissue areas. Only the tissue area of the ONH was cross-sectionally analyzed for the pulse waveform analysis. Moreover, the LSFG analyzer software enables the recording of synchronized images from each cardiac cycle (Fig. 1B, C) and determines the values of various heartbeat waveform parameters. Eight pulse waveform parameters were evaluated, which include the skew, blowout score (BOS), blowout time (BOT), rising rate (RR), falling rate (FR), flow acceleration index (FAI), acceleration time index (ATI), and resistivity index (RI) (Fig. 3).

The eight pulse waveform parameters are calculated with the following equation:

Skew= $$ \mathrm{C}\bullet {\int}_0^N{\left\{\left(x-\mathrm{Ave}\right)/\mathrm{Stdev}\right\}}^3\bullet p(x)\mathrm{dx} $$

Ave= $$ {\int}_0^Nx\bullet p(x)\mathrm{dx} $$

Stdev= $$ \sqrt{\int_0^N{\left(x-\mathrm{Ave}\right)}^2\bullet p(x)\mathrm{dx}} $$

Where C is the constant of proportion, the variable N represents the frame number, and the changing mean blur rate (MBR) waveform of a beat is divided into N frames. The variable m(k) is the average of MBR in the k-th frame. Max represents the maximum MBR value and min is the minimum MBR value.

BOS= $$ \mathrm{C}\bullet \left[\left\{2-\left({MBR}_{max}-{MBR}_{min}\right)/\mathrm{mean}\right\}/2\right] $$

$$ \mathrm{mean}=\left({\sum}_{k=1}^{\mathrm{N}}m(k)\right)/\mathrm{N} $$

$$ \mathrm{BOT}=\mathrm{C}\bullet \left\{{\sum}_{k=1}^{\mathrm{N}}h(k)\right\}/\mathrm{N} $$

$$ h(k)=\left\{\begin{array}{c}1\kern4.25em m(k)>\left({MBR}_{max}+{MBR}_{min}\right)/2\\ {}0\kern4em \mathrm{otherwise}\kern11em \end{array}\right. $$

$$ \mathrm{RR}=\mathrm{C}\bullet \left\{\left({\sum}_{k=1}^{f_{max}}m(k)\right)-{MBR}_{min}\bullet {f}_{max}\right\}/\left\{\left({MBR}_{max}-{MBR}_{min}\right)\bullet {f}_{max}\right\} $$

$$ \mathrm{FR}=\mathrm{C}\bullet \left\{{MBR}_{max}\bullet \left(\mathrm{N}-{f}_{max}+1\right)-{\sum}_{k={f}_{max}}^{\mathrm{N}}m(k)\right\}/\left\{\left({MBR}_{max}-{MBR}_{min}\right)\bullet \left(\mathrm{N}-{f}_{max}+1\right)\right\} $$

FAI = MAX {*m*(*k* + 1) − *m*(*k*)} k = 1, 2, …,N-1

$$ \mathrm{ATI}=100\bullet {f}_{max}/\mathrm{N} $$

$$ \mathrm{resistivity}\ \mathrm{index}=\left({MBR}_{max}-{MBR}_{min}\right)/{MBR}_{max} $$

The LSFG was measured two times at each time point using the follow-up mode in all of the eyes. The average of values was used for the statistical analyses.

### Statistical analyses

We evaluated the pulse waveforms using a linear mixed model to incorporate possible correlations between repeated measured values of the parameters for each eye over time within a subject, which was the same statistical method as previously reported [1, 2]. Briefly, we assumed the following model,

*y*_*ij*_ = *a*_*i*_ *+ f (tj:b) + ε*_*i j*_*.*

*i* (subject) = 1, ….,10, *j* (time) = before, 0, 1, 3, 5, 7, 10, 11, 13,15 (min) where y_*ij*_ is the pulse waveform parameters at time j of subject i and *a*_*i*_ is a subject-specific random effect. The function *f (tj:b)*, which represents a fixed effect of time on the refraction, was specified as a polynomial function. The *b* parameters represent the fixed time effects and interaction between the time and group effects, respectively. The order of polynomials in *f (tj:b)* was selected on the basis of the Akaike information criteria.

The level for statistical significance was 0.05. The statistical analyses were performed with SAS9.3 MIXED procedure (SAS Inc., Cary).

## Results

### Subjects’ dispositions, demographics, and baseline characteristics

For the two experiments, 30 healthy Japanese individuals were recruited, and the demographics of the participating volunteers are shown in Tables 1 and 2 for experiments 1 and 2, respectively. Two subjects could not complete the examination and dropped out of experiment 1 because they could not tolerate the application of the ophthalmodynamometer on the sclera. Additionally, two subjects were excluded from experiment 2 because their SBP was > 150 mmHg. In the end, 16 volunteers with an average age of 33.1 ± 8.6-years completed all phases of the examinations in experiment 1. Ten volunteers with an average age of 28.6 ± 1.0-years completed all phases of experiment 2. No adverse events were observed during and after the measurements in any of the participants.

**Table 1:** Baseline Characteristics of Subject (Experiment 1)

Characteristics (***n*** = 16)	mean ± SD
Age (years)	33.1 ± 8.6
IOP (mmHg)	15.2 ± 3.0
Axial length (mm)	25.7 ± 0.96
Refractive error (diopter)	−4.19 ± 2.60
Systolic blood pressure (mmHg)	116.9 ± 14.3
Diastolic blood pressure (mmHg)	72.6 ± 11.0
Heart rate (BPM)	70.1 ± 6.9

**Table 2:** Baseline Characteristics of Subject (Experiment 2)

Characteristics (***n*** = 10)	mean ± SD
Age (years)	28.6 ± 1.0
IOP (mmHg)	12.7 ± 2.5
Axial length (mm)	25.8 ± 1.16
Refractive error (diopters)	−4.84 ± 2.74
Systolic blood pressure (mmHg)	110.6 ± 7.9
Diastolic blood pressure (mmHg)	67.7 ± 6.6
Heart rate (BPM)	72.6 ± 10.4

### Changes in IOP and OPP in experiment 1

A stable and significant increase in the IOP by 30 mmHg was caused by the application of the ophthalmodynamometer for 10 min (*P* < 0.001; Fig. 5a), and the MAP did not change significantly (Fig. 5b). As a result, the OPP decreased significantly during the IOP elevation (Fig. 5c). After the release of the pressure, the IOP and OPP returned to pressures that did not differ significantly from the baseline pressures. No significant change in the heart rate was observed during the experiment.

### Changes in pulse waveform parameters on ONH in experiment 1

The MBR reduced significantly from 11.9 ± 1.9 AU to 9.8 ± 2.3 AU (− 18.3%) after increasing the IOP by 20 mmHg and to 9.1 ± 2.5 AU (− 25.0%) after increasing the IOP by 30 mmHg (both, *P* < 0.001), and returned to 12.2 ± 2.7 AU after the release of the pressure. The IOP returned to the baseline after the pressure was released.

**Fig. 4:**
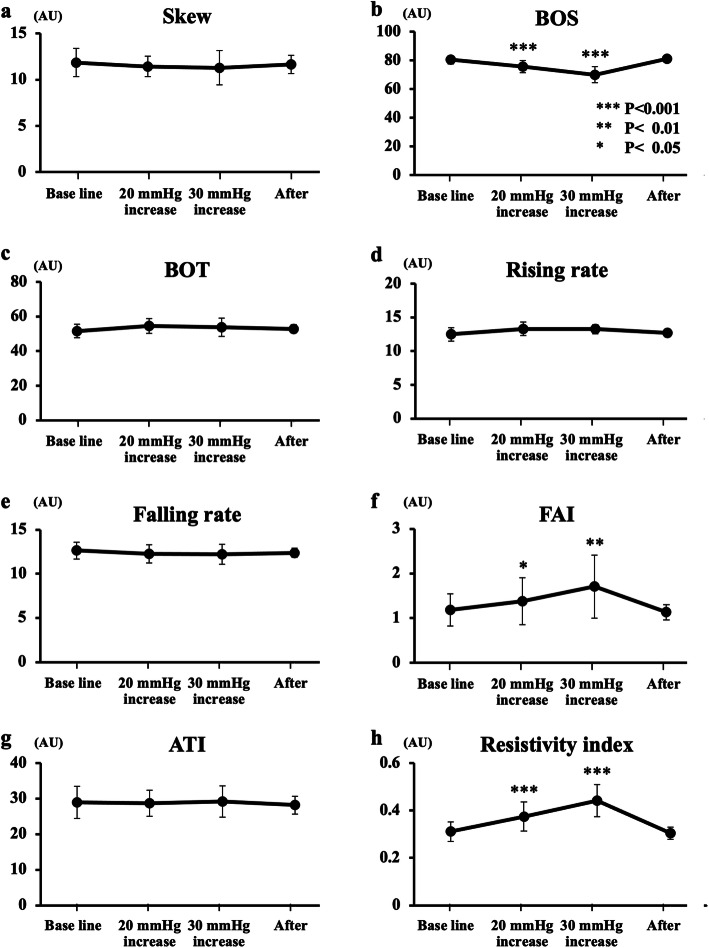
Changes in the eight waveform parameters of the blood flow on the ONH are shown (**a**-**h**). Of the eight pulse waveform parameters, the BOS (**b**) was significantly decreased during IOP elevation, and the FAI (**f**) and the resistivity index (**h**) were significantly increased after the elevation of the IOP by 20 and 30 mmHg, respectively. The values of three parameters returned to the baseline after IOP release. No significant changes were observed in the Skew (**a**), BOT (**c**), rising rate (**d**), falling rate (**e**) and ATI (**g**) after the IOP elevation. *** *P* < 0.001, ** *P* < 0.01, * *P* < 0.05

Of the eight waveform parameters, three parameters mentioned below changed significantly after the IOP elevation and they recovered to their baseline after the release of the pressure (Fig. 4). The BOS (*P* < 0.001) decreased significantly, and the FAI (*P* < 0.01) and RI (*P* < 0.001) increased significantly. No significant change was observed in the Skew, BOT, RR, FR, and the ATI after the IOP elevation by both 20 and 30 mmHg.

### Changes in IOP and OPP in experiment 2

**Fig. 5:**
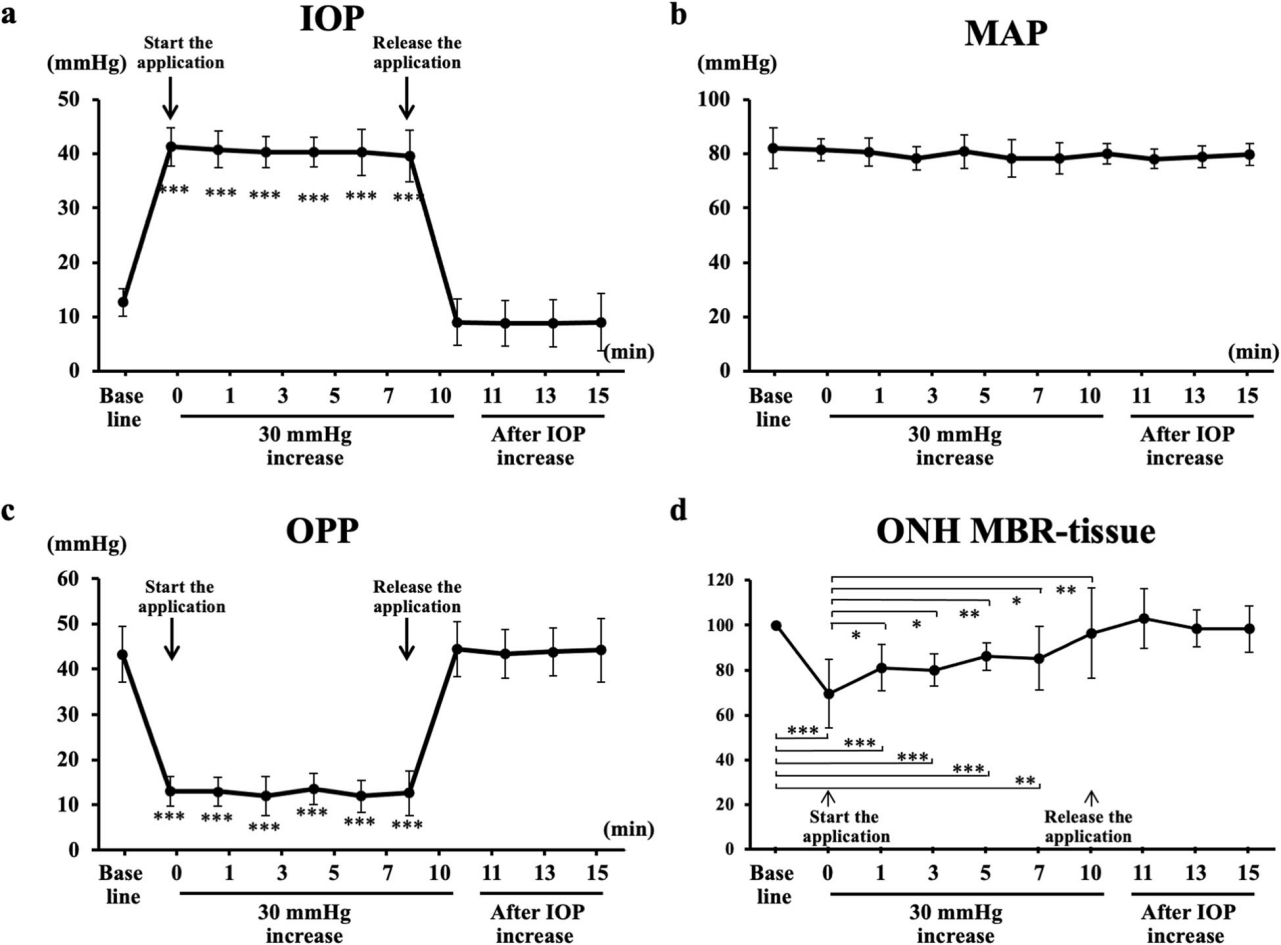
Changes in the systemic and ocular parameters before IOP increased, when 30 mmHg IOP increased for 10 min, and after IOP returned to baseline pressure. The IOP significantly increased (**a**), the MAP was not changed (**b**), and the OPP (**c**) significantly decreased during the application of the pressure, using the ophthalmodynamometer. The MBR was significantly reduced immediately after the IOP elevation and remained significantly reduced until 7 min after the IOP elevation (**d**). The MBR significantly increased from time 1 through time 10 compared to time 0. After the release of the pressure, the MBR returned to the baseline level immediately. *** *P* < 0.001, ** *P* < 0.01, * *P* < 0.05

A stable and significant increase in the IOP by 30 mmHg was caused by the application of the ophthalmodynamometer for 10 min (*P* < 0.001; Fig. 5a), and the MAP did not change significantly (Fig. 5b). As a result, the OPP decreased significantly during the IOP elevation (Fig. 5c). After the pressure was released, the IOP and OPP returned to pressures that did not differ significantly from the baseline pressures. No significant change in the heart rate was observed during the experiment.

### Changes in pulse waveform parameters on ONH in experiment 2

The MBR reduced significantly from 10.4 ± 2.0 AU to 7.4 ± 2.3 AU immediately after the 30-mmHg IOP elevation (time 0) and remained significantly reduced until 7 min after the IOP elevation compared to the baseline (*P* < 0.001, Fig. 5d). During the 30-mmHg IOP elevation, the MBR increased significantly from time 1 (*P* < 0.05) to time 10 (*P* < 0.01) compared to time 0 (immediately after IOP elevation). After the release of the pressure, the MBR returned to the baseline level.

**Fig. 6:**
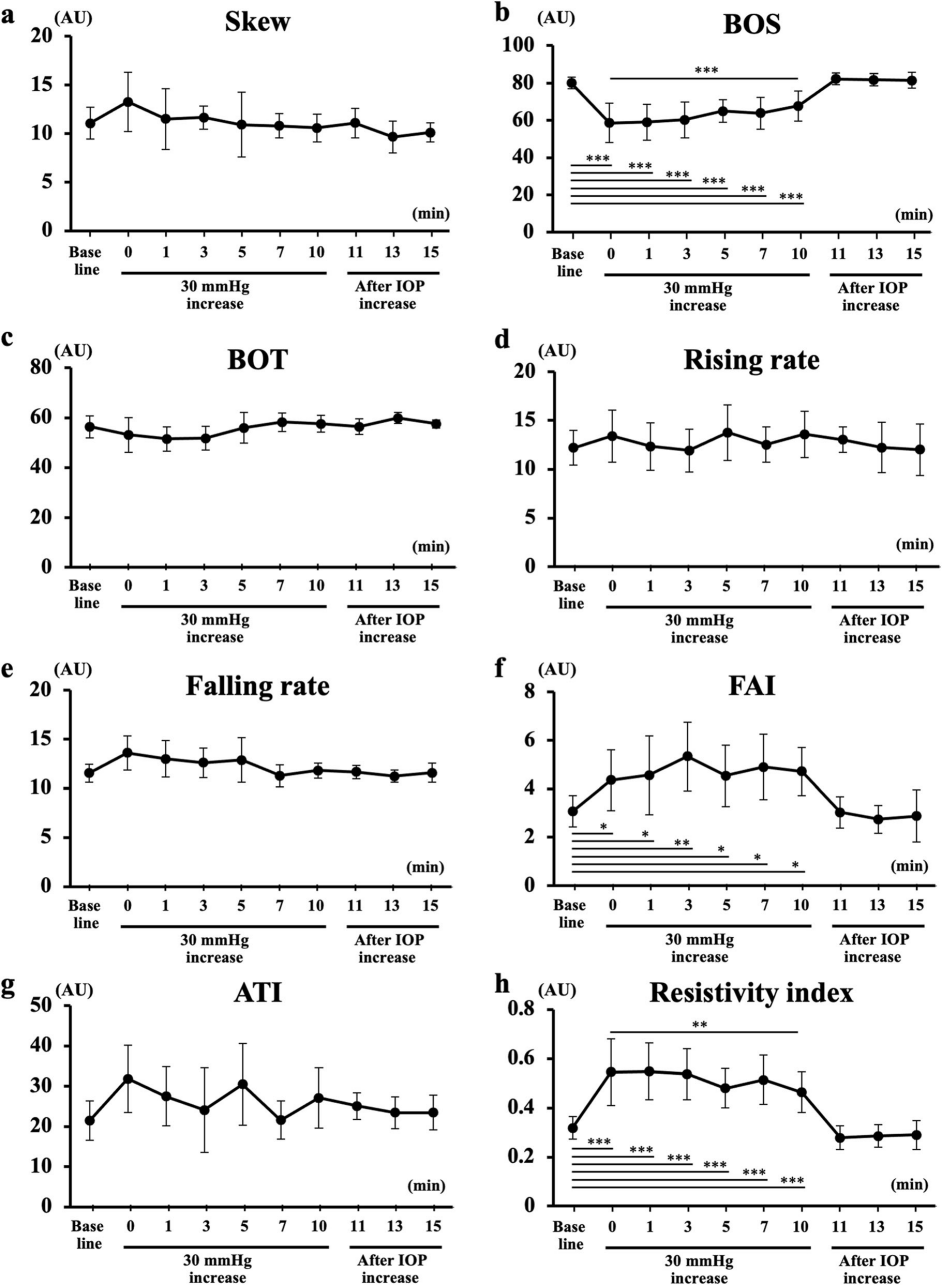
The changes in the eight waveform parameters on the ONH are shown. The BOS was significantly reduced during the IOP elevation throughout the 10 min, and the FAI and RI significantly increased. Of the three parameters with significant changes during the IOP elevation, the BOS and RI significantly returned to the baseline level at time 10 compared to that at time 0

The changes in the eight waveform parameters on the ONH are shown in Fig. 6. The BOS reduced significantly after the IOP elevation throughout the 10 min (*P* < 0.001), and the FAI (*P* < 0.001) and RI (*P* < 0.001) increased significantly compared to the baseline (before IOP elevation). Of the three parameters with significant changes during the IOP elevation, the degree of decrease in the BOS and increase in the RI at time 10 decreased significantly compared to those at time 0 (immediately after IOP elevation) (*P* < 0.001 and *P* = 0.008, respectively). All three parameters returned to the baseline after the pressure was released.

No significant changes in the skew, BOT, RR, FR, and ATI were observed during the IOP elevation.

**Fig. 7:**
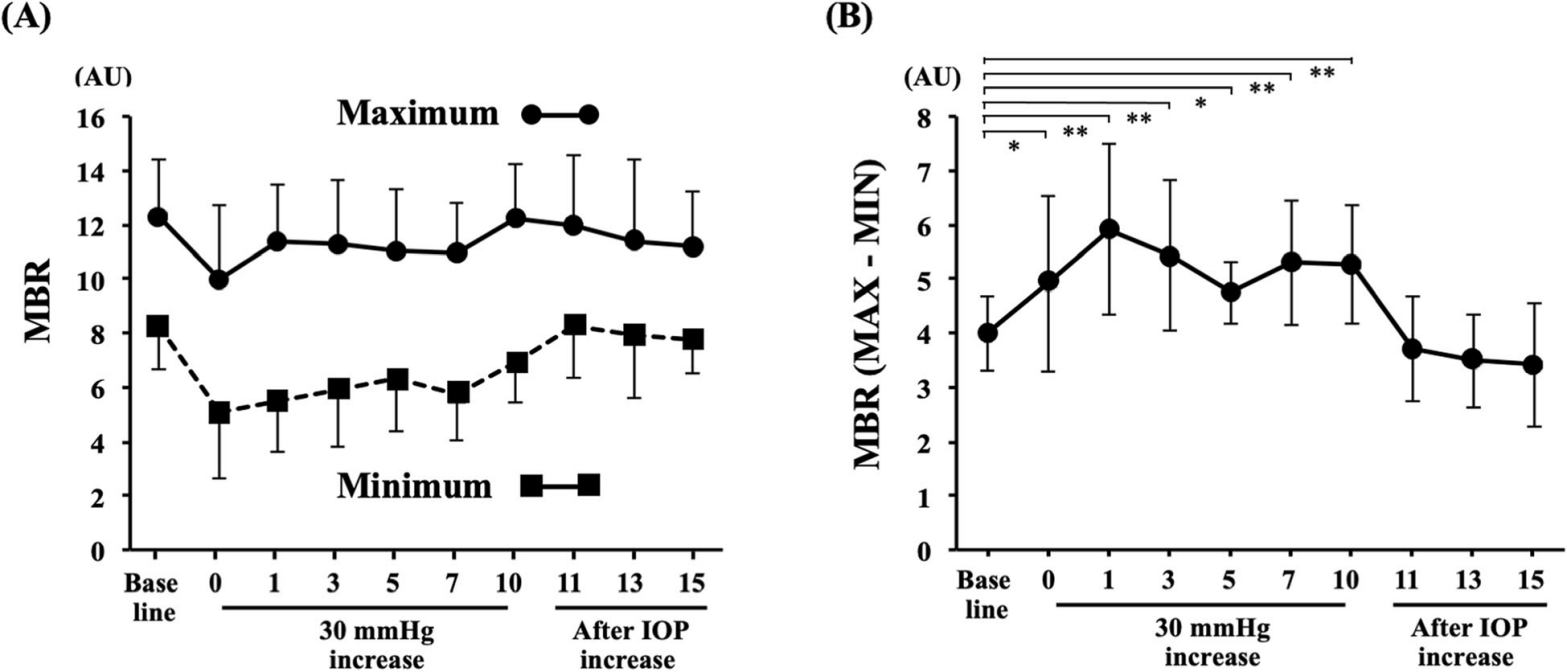
The changes in the maximum and minimum MBR (**a**) and in the difference between the maximum and minimum MBR are shown (**b**). The difference between the maximum and minimum MBR significantly increased during the IOP elevation. ** *P* < 0.01, * *P* < 0.05

The changes in the mean maximum and minimum MBRs in a heartbeat are shown in Fig. 7. The difference between the maximum and minimum MBRs was significantly greater at some time points during the IOP elevation.

## Discussion

This study investigated the influences of IOP elevations on the pulse waveform parameters determined using LSFG in the ONH evaluated in normal subjects. Our study had two arms. In experiment 1, the pulse waveform parameters were measured during the transient IOP elevation by 20 and 30 mmHg from the baseline. In experiment 2, the pulse waveform parameters were measured during the 10-min IOP elevation by 30 mmHg from the baseline. The results showed that the BOS, FAI, and RI of the pulse waveforms changed significantly in response to changes in the OPP induced by an elevation of the IOP by 20 or 30 mmHg. The three parameters changed significantly during the 10-min IOP elevation by 30 mmHg from the baseline, and the degree of decrease in the BOS and increase in the RI at time 10 decreased significantly compared to those at time 0 during the IOP elevation in experiment 2. Additionally, the difference between the maximum and minimum MBRs was significantly greater during the IOP elevation.

The BOS demonstrates the constancy of the blood flow during a beat, and the RI indicates the resistance to flow in the vessels. Considering the formula to calculate the BOS and RI, the greater difference between the maximum and the minimum MBR becomes a lower BOS and a higher RI during IOP elevation, and these parameters should have a strong inverse relationship [20, 32]. Our results showed the opposite changes in the two waveforms, for example, the BOS reduced and the RI increased after IOP elevation. Additionally, the degree of the BOS decrease and the RI increase was greater with the higher elevation of the IOP in experiment 1. These results indicate clearly that blood flow on the ONH is suppressed because of the higher resistance to flow by IOP elevation.

Kiyota et al. reported similar significant changes in these parameters, the BOS, RI, and FAI on the ONH and the choroid after artificial experimental IOP elevation [33]. Pappelis et al. also reported that the RI increased significantly after IOP elevation [34]. Moreover, Takeshima et al. reported the changes in the MBR after the IOP decrease with glaucoma surgery trabeculectomy, which was an inverse change in IOP when compared to our experiment [32]. Their results showed the inverse response as a significant increase in the BOS and a significant decrease in the RI. Our results corroborate those previous reports [32–34]. These results using LSFG are in good agreement with the earlier color Doppler imaging-derived findings that the retrobulbar central retinal artery blood flow velocity decreased and the RI increased during the IOP elevation [35].

Interestingly, our results have shown that the difference between the maximum and minimum MBRs became greater during the IOP elevation. Considering the characteristics of the MBR, the maximum and minimum MBRs would reflect responses of the artery and the vein in a beat, respectively. The response of the retinal arterial and venous diameters to an experimental increase in the IOP has been reported to be different [36, 37]. The arteries are dilated in response to increased IOP, which is an autoregulatory response to keep the blood flow constant, but the diameter of veins decreases because the veins probably reflect a passive compression effect owing to the weaker wall construction of the retinal veins than that of the arteries [36, 37]. The difference of response between the artery and the vein would result in a greater difference for experimental IOP elevation.

The degree of decrease in the BOS and increase in the RI at time 10 decreased significantly compared to those at time 0 (immediately after IOP elevation) during the IOP elevation. Conversely, the IOP and the OPP did not change. These results suggest that the decreased blood flow recovered gradually even during stable IOP elevation, because the increased resistance was decreased. This response implies the presence of an autoregulatory response to supply blood flow even though the OPP remained reduced in our experiment.

The ATI was derived from a ratio of the length of time before reaching the peak to the length of time for the entire heartbeat. A lower ATI indicates a more rapid increase in the MBR to the peak. Our result showed that the ATI did not change after the IOP elevation. This result is in good agreement with the previous report [33]. In the present study, the ATI and heart rate did not change after the IOP elevation, meaning that the time to the peak in a beat did not change after the IOP elevation.

**Fig. 8:**
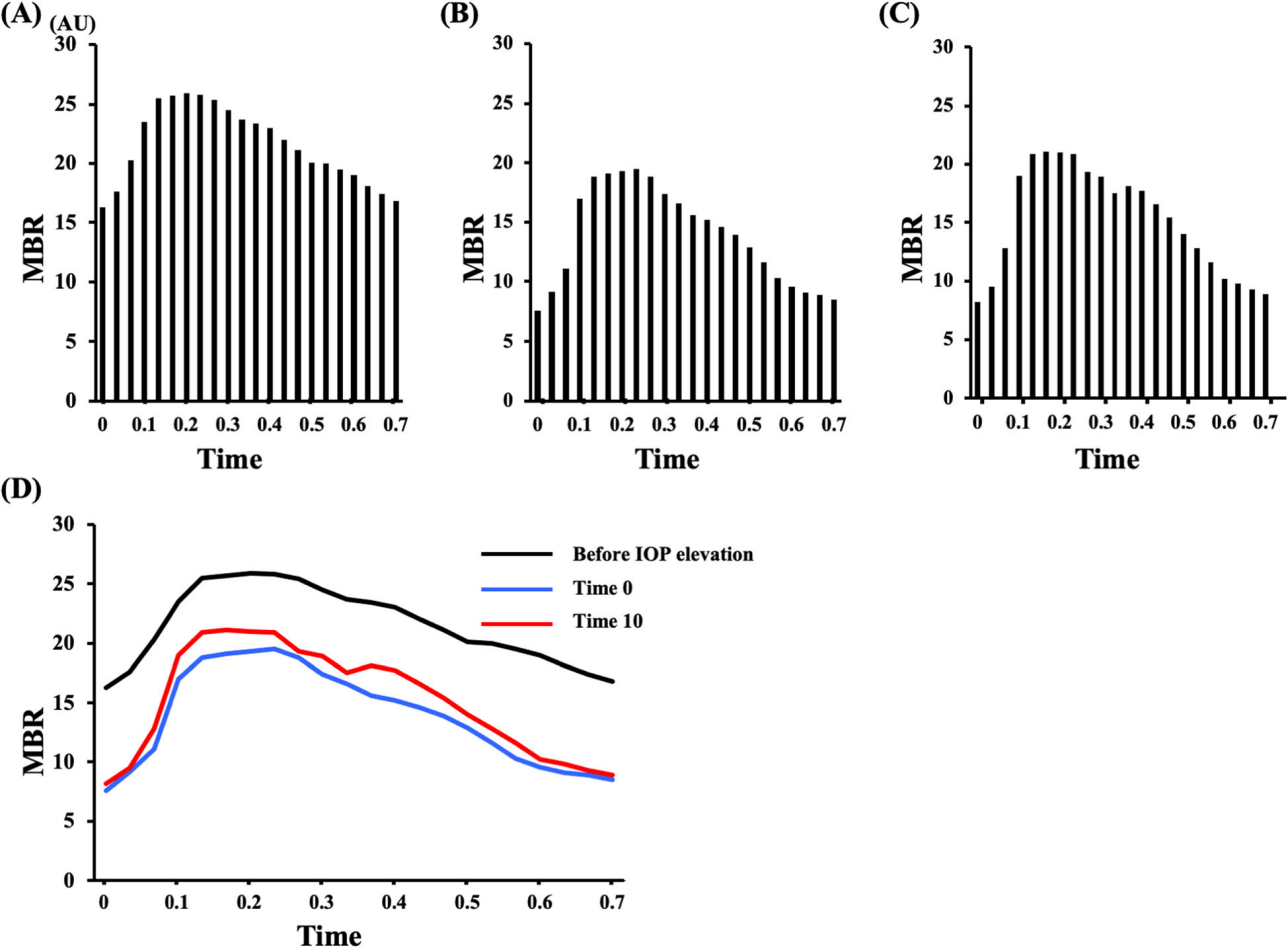
Representative MBR pulse waveforms on the ONH before (**a**), immediately (time 0) (**b**), and 10 min after the IOP elevation (time 10) (**c**). Additionally, a comparison highlighting the changes that occurred over a heartbeat is shown (**d**). The observed MBR after IOP elevation was lower throughout the time of a heartbeat than that before the IOP elevation, but the difference at the peak of the MBR (maximum MBR) between before and after the IOP elevation was relatively lower than that in the bottom of the MBR (minimum MBR). Additionally, MBR pulse waveform at time 10 was higher than that at time 10 in a heartbeat

Representative MBR changes in a beat of the ONH before (a), immediately after (b), and 10 min after the IOP elevation are shown in Fig. 8. These changes show that the MBR reduced throughout a beat during IOP elevation. Moreover, as mentioned above, the minimum MBR was more reduced than the maximum MBR, resulting in a large difference between the maximum and minimum MBRs, and the peak did not change after the IOP elevation.

The FAI increased during the IOP elevation, and it was calculated from the maximum change of all frames (1/30 s) in a rising curve [20, 38]. This result should be because of the large difference between the maximum and minimum MBRs and not a significant difference in time reaching the peak in a heartbeat. This result is in good agreement with the previous report [33, 34].

The skew quantifies the asymmetry of the waveform distribution, varying with the bias of the waveform shape. It is an indicator of asymmetry of the MBR waveform. A negative value indicates a rightward shift of the MBR waveform and/or a gentler slope after the peak. Our results showed a significant difference between the maximum and minimum MBRs and no difference in time to the peak of a heartbeat. This result should cause a gentler slope after the peak, leading a negative value of the skew from the calculation. However, the skew did not change after the IOP elevation in our experiments. Conversely, previous reports have shown that the skew was reduced significantly after the IOP elevation [33, 34]. The exact reasons for the difference between their results and ours are unknown, but one of the reasons may be the sample size.

The BOT is the ratio of the duration of the MBR being maintained for more than half of the mean of the maximum and minimum MBRs to the duration of one heartbeat. A higher BOT indicates that a high level of MBR is maintained for a larger proportion of a single heartbeat. The FR is defined as the ratio of the falling area to the total area after the peak. A higher FR value indicates a more sudden decrease in the MBR. A gentler slope after the peak should cause a lower BOT and FR from the calculation. Actually, a previous report has shown a lower BOT and FR after the IOP elevation [33]. However, the degree of change in the skew, BOT, and FR were lower than that in the BOS, RI, and FAI in their results [33]. Elucidating the actual reasons of some differences between their results and ours by increasing the sample size.

The strength of our study is that the change in pulse waveforms was evaluated during a relatively long period of 10 min. A transient response for the acute elevation of the IOP has been examined in previous reports [32–34]. The degree of significant change in the BOS, RI, and FAI decreased during 10 min in our results, indicating the presence of some autoregulation on the ONH.

This study has several limitations. First, the variables were measured only with 20- and 30-mmHg elevations. Several studies have reported on the effects of stepwise elevations of the IOP [15, 39–43]. Second, the ONH blood flow largely recovered probably because of autoregulation after 10 min of the IOP elevation, but it did not return completely to the baseline levels. A longer period of IOP elevation measurements is needed to determine whether the ONH blood flow recovers fully. However, the application of pressure for durations longer than 10 min was painful, and it could not be extended for ethical reasons. Third, our study participants had several myopic eyes. The morphological features of the optic disc of myopic and nonmyopic eyes are different [44], which might affect the results. Fourth, only relatively young subjects were studied, and the results cannot be extrapolated to elderly subjects. Fifth, our sample size was relatively small. Further studies with a larger number of subjects, including nonmyopic subjects, a wider range of ages, and IOP elevations in a step-by-step manner, are needed.

## Conclusion

In conclusion, the BOS, FAI, and RI of the pulse waveforms on the ONH-tissue changed significantly by an acute elevation of the IOP. The change should be related to the greater difference between the maximum and minimum MBRs during the IOP elevation.

## Data Availability

The datasets generated during and analyzed during the current study are not publicly available due to privacy and ethical concerns but are available from the corresponding author on reasonable request.

## Abbreviations

ONH: optic nerve head
IOP: intraocular pressure
LSFG: laser speckle flowgraphy
MBR: mean blur rate
OPP: ocular perfusion pressure
BOS: blowout score
FAI: flow acceleration index
RI: resistivity index
SBP: systolic blood pressure
DBP: diastolic blood pressure
MAP: mean arterial blood pressure
BOT: blowout time
RR: rising rate
FR: falling rate
ATI: acceleration time index
